# Cinema Theatres and Infectious Diseases

**Published:** 1913-01-11

**Authors:** 


### Cinema Theatres and Infectious Diseases.

*To the Editor of* The Hospital.

Sir,—I was glad to see from your note in The Hospital last week that Dr. Jones, County Medical Officer for Surrey, has also drawn attention to the risk of spreading such diseases as scarlet fever, measles, chicken-pox, etc., which exists in connection with the present popularity of cinematograph theatres. I think there is little doubt that numerous cases of infectious disease are traceable to this source. Although actual verification of this statement is difficult, I, in common with other medical officers of fever hospitals, have often found that on going into the history of a case the most probable place of infection was a cinema theatre.

It would be interesting to compare the figures, say, of measles or scarlet fever incidence during school terms and during holiday time, when perhaps the attendance of children at these places would be augmented by their greater leisure. The exclusion of light from these halls—often continuously and always for many hours daily—would of course greatly favour the continuance of the activity of the germs scattered about, whereas sunlight would speedily cause their death.—I am, yours, etc.,

January 6. Medical Superintendent.

